# Postoperative Trends and Prognostic Values of Inflammatory and Nutritional Biomarkers after Liver Transplantation for Hepatocellular Carcinoma

**DOI:** 10.3390/cancers13030513

**Published:** 2021-01-29

**Authors:** Riccardo Pravisani, Federico Mocchegiani, Miriam Isola, Dario Lorenzin, Gian Luigi Adani, Vittorio Cherchi, Maria De Martino, Andrea Risaliti, Quirino Lai, Marco Vivarelli, Umberto Baccarani

**Affiliations:** 1 Liver-Kidney Transplantation Unit, Department of Medicine, University of Udine, 33100 Udine, Italy; dario.lorenzin@asufc.sanita.fvg.it (D.L.); gianluigi.adani@asufc.sanita.fvg.it (G.L.A.); vittorio.cherchi@asufc.sanita.fvg.it (V.C.); andrea.risaliti@uniud.it (A.R.); umberto.baccarani@uniud.it (U.B.); 2 HPB Surgery and Transplantation Unit, Department of Clinical and Experimental Medicine, Polytechnic University of Marche, 60126 Ancona, Italy; Federico.Mocchegiani@ospedaliriuniti.marche.it (F.M.); marco.vivarelli@ospedaliriuniti.marche.it (M.V.); 3 Division of Medical Statistic, Department of Medicine, University of Udine, 33100 Udine, Italy; miriam.isola@uniud.it (M.I.); maria.demartino@uniud.it (M.D.M.); 4 Hepato-biliary and Organ Transplant Unit, Department of Surgery, Sapienza University of Rome, 00161 Rome, Italy; lai.quirino@libero.it

**Keywords:** controlling nutritional status, prognostic nutritional index, platelet-to-lymphocyte ratio, neutrophil-to-lymphocyte ratio, hepatocellular carcinoma, liver transplantation

## Abstract

**Simple Summary:**

Inflammatory biomarkers have a strong prognostic value in surgically treated patients with hepatocellular carcinoma (HCC), but the underlying pathogenic mechanism has not been completely clarified. Conversely, nutritional biomarkers predict the outcomes after hepatic resection for HCC but not after liver transplantation (LT). Indeed, the impact of LT on the recipient’s nutritional status is heterogeneous, while the data on the patient’s outcome after LT in terms of inflammatory status are limited. Therefore, to address these unsolved questions, we conducted a retrospective analysis on 324 HCC patients treated with LT, exploring the postoperative trend up to 1 year post-LT and the prognostic value of the Platelet-to-Lymphocyte Ratio (PLR), Neutrophil-to-Lymphocyte Ratio (NLR), Controlling Nutritional Status (CONUT), Prognostic Nutritional Index (PNI). It was found that at 1 year post-LT, the nutritional status of liver-transplanted HCC patients significantly improved while their inflammatory state tended to persist. Consequently, post-LT PLR and NLR maintained a prognostic value for LT outcome while post-LT CONUT and PNI acquired it.

**Abstract:**

Preoperative inflammatory biomarkers such as the Platelet-to-Lymphocyte Ratio (PLR) and the Neutrophil-to-Lymphocyte Ratio (NLR) strongly predict the outcome in surgically treated patients with hepatocellular carcinoma (HCC), while nutritional biomarkers such as the Controlling Nutritional Status (CONUT) and the Prognostic Nutritional Index (PNI) show an analogue prognostic value in hepatic resection (HR) but not in liver transplant (LT) cases. Data on the impact of LT on the inflammatory and nutritional/metabolic function are heterogeneous. Therefore, we investigated the post-LT trend of these biomarkers up to postoperative month (POM) 12 in 324 HCC patients treated with LT. Inflammatory biomarkers peaked in the early post-LT period but at POM 3 leveled off at values similar (NLR) or higher (PLR) than pre-LT ones. CONUT and PNI worsened in the early post-LT period, but at POM 3 they stabilized at significantly better values than pre-LT. In LT recipients with an overall survival >1 year and no evidence of early HCC recurrence, 1 year post-LT NLR and PNI independently predicted patient overall survival, while 1 year post-LT PLR independently predicted late tumor recurrence. In conclusion, at 1 year post-LT, the nutritional status of liver-transplanted HCC patients significantly improved while their inflammatory state tended to persist. Consequently, post-LT PLR and NLR maintained a prognostic value for LT outcome while post-LT CONUT and PNI acquired it.

## 1. Introduction

Laboratory-derived clinical biomarkers have been recently shown to provide objective, measurable and synthetic information on the clinical status of the patient [1,2]. Moreover, they seem to have a prognostic value for some patient outcomes [1,2,3,4,5,6]. In patients with hepatocellular carcinoma (HCC) treated with hepatic resection (HR) or liver transplantation (LT), the Neutrophil-to-Lymphocyte Ratio (NLR) and the Platelet-to-Lymphocyte Ratio (PLR) have been validated as markers of systemic inflammation and immune dysfunction, and when tested preoperatively, they have been shown to predict the risk of early death or tumor recurrence [2,3,4,5,6,7,8]. On the other hand, the Controlling Nutritional Status (CONUT) score and the Prognostic Nutritional Index (PNI), that are validated nutritional biomarkers, have shown an analogue prognostic value in HR cases but not in the LT setting [9,10,11,12]. The underlying pathogenic mechanism associated with such diversity is currently not known, as very limited data are available on the effect of LT on the immuno-inflammatory and nutritional status of the recipient. Therefore, the present study explores the postoperative trend and prognostic value of PLR, NLR, CONUT, and PNI, aiming at (i) providing new insights on the impact of LT on the recipient’s inflammatory and nutritional status, (ii) trying to explain the diversified prognostic value of inflammatory and nutritional biomarkers as preoperative variables, and (iii) testing whether these biomarkers can be used also as postoperative surveillance parameters.

## 2. Materials and Methods

This is a retrospective study on a multicenter cohort of 324 HCC patients treated with LT at the Liver-Kidney Transplant Unit—Udine University Hospital (*n* = 126), and at the HPB Surgery and Transplantation Unit—United Hospitals of Ancona (*n* = 198), between January 2006 and December 2018. Demographic and clinical data of recipients and intraoperative and postoperative outcomes were reviewed from the local electronic database. The pre-transplant assessment was previously described in [10]. CONUT score was calculated as previously described [10], while PNI was calculated according the following formula [10 * serum albumin (g/dL) + 0.005 * Total lymphocyte (cells/mm2)] (PNI). CONUT score, Model for end-stage liver disease (MELD) score, PNI, PLR, and NLR, were calculated on laboratory test data on admission before LT. As postoperative variables, PLR, NLR, CONUT, and PNI were calculated at postoperative day (POD) 1, 3, 5, and 7, and at postoperative month (POM) 3, 6, and 12. Standard post-transplant management for clinical surveillance over postoperative complications as well as immunosuppressant regimens and post-discharge patient follow-up protocols were previously described in [10].

Categorical variables were expressed by frequencies and percentage, while continuous variables were expressed by mean ± standard deviation (SD) or median (interquartile range (IQR)), as appropriate. General Linear Model for measured repeats was used to compare post-LT values of PLR, NLR, PNI, and CONUT at determined time points, with their respective pre-LT values, after the assumptions had been verified. Bonferroni’s correction for multiple comparisons was applied. Overall survival (OS) was defined as the time (months) from liver transplantation to either death or last observation and was described according to the Kaplan–Meier approach. Univariate Cox regression was used to estimate prognostic value of pre-LT and post-LT biomarkers for OS, after the assumption of the proportional hazard was verified. The proportional hazard assumption was tested using the Schoenfeld residual test. Death was considered as a competing risk event because death for causes unrelated to HCC precludes the occurrence of HCC recurrence. The cumulative incidence method was used to estimate HCC recurrence accounting for the presence of competing risks. Based on the method of Fine and Gray, univariate competing-risk regression was used to explore whether pre- and post-LT biomarkers were associated with HCC recurrence. The competing-risk regression model is based on the hazard of the subdistribution and provides a simple relationship between covariates and cumulative incidence. In case of HCC recurrence within 1 year post-LT (early recurrence), later biomarker data after recurrence were excluded from the analysis.

To test the surveillance value as prognostic factor of 1-year post-LT inflammatory and nutritional biomarkers, a sub-group analysis was performed in patients with an OS > 1 year and no evidence of early HCC recurrence (*n* = 266). A univariate and stepwise multivariate Cox regression was used for OS analysis, while a univariate and stepwise multivariate competing-risk regression was used for HCC recurrence analysis. The risk of multicollinearity was evaluated by means of the variance inflation factor. Variables of p less than 0.10 during univariate analysis were included in multivariable analysis.

Univariate linear regression model was used to explore the potential determinants of prognostically significant biomarkers among HCC features, MELD score, and Child–Pugh classification.

The analyses were performed using Stata/SE 15.1 (Stata Corp LP, United States). The present study was approved by the local Institutional Review Board.

## 3. Results

The demographic and clinical data on recipients, HCC histopathology characteristics, surgical details, and donor and graft characteristics are summarized in Table 1. The median age at transplantation was 58 years (52–62) with a median MELD score of 12 (9–16) and median BMI of 25.3 (23.2–28.1). At pathologic examination of the explanted liver, the median number of HCC lesions was 2 (1–3) with bilobar distribution in 25.3% of cases and a median maximum diameter of 2.3 (1.5–3) cm. The median follow-up time was 50.2 months (21.9–91.4).

### 3.1. Post-Transplant Trend of Inflammatory and Nutritional Biomarkers

**Figure 1:**
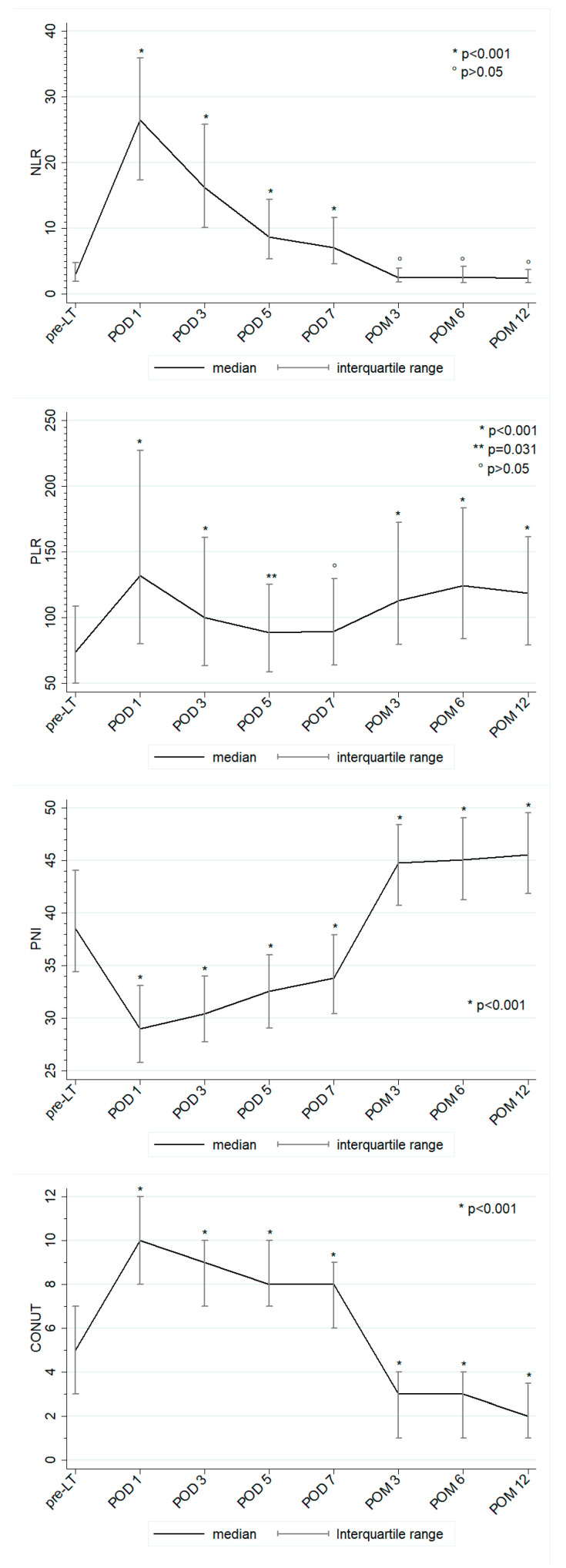
Postoperative trend of PLR, NLR, PNI, and CONUT. *p* values refer to the confrontation between post-LT and pre-LT biomarker values.

The preoperative values of PLR, NLR, PNI, and CONUT were 74.2 (50.3–108.5), 2.9 (1.9–4.8), 38.6 (34.5–44.1), and 5 (3–7), respectively. The postoperative trend of these scores is shown in Figure 1. Compared to preoperative values, the nutritional biomarkers showed a significant worsening in the early post-LT period (Figure 1, decrease of PNI and increase of CONUT), followed by a significant improvement at POM 3 which tended to stabilize thereafter for CONUT while further significantly improving for PNI. At 1-year post-LT, the median value of PNI and CONUT were 45.8 (41.9–49.5) and 2 (1–4). Both PLR and NLR peaked on POD 1 and subsequently decreased, still maintaining values significantly higher than pre-LT ones. At POM 3, both inflammatory biomarkers leveled off at values that were similar to pre-LT for NLR (1-year post-LT value 2.4 [1.7–3.7], *p* 0.328) but significantly higher for PLR (118.2 [79.1–161.5], *p* < 0.001).

### 3.2. Overall Survival

The overall patient survival at 1, 3, and 5 years was 85.6%, 76.1%, and 70.1%, respectively. In univariate analysis (Table 2), Pre-LT PLR, pre-LT NLR, but not pre-LT PNI nor pre-LT CONUT were confirmed as significant prognostic factors, as previously reported [10]. Moreover, analyzing the inflammatory and nutritional biomarkers as postoperative values, NLR and PLR maintained a stable significant prognostic value from POD 3 and POD 7, respectively, while PNI and CONUT acquired a stable prognostic value since POD 7.

### 3.3. HCC Recurrence

The cumulative incidence of HCC recurrence at 1, 3, and 5 years was 4.4%, 12.2%, 15.8%, respectively. In univariate analysis (Table 2), pre-LT PLR, pre-LT NLR, as well as post-LT PLR since POM 6, NLR since POM 3, and PNI at POM 12, predicted tumor recurrence.

### 3.4. Late (>1 Year Post-LT) Outcomes

We further investigated the potential predictive factors for OS and HCC recurrence in patients with an OS > 1 year and no evidence of early HCC recurrence (*n* = 266).

**Figure 2:**
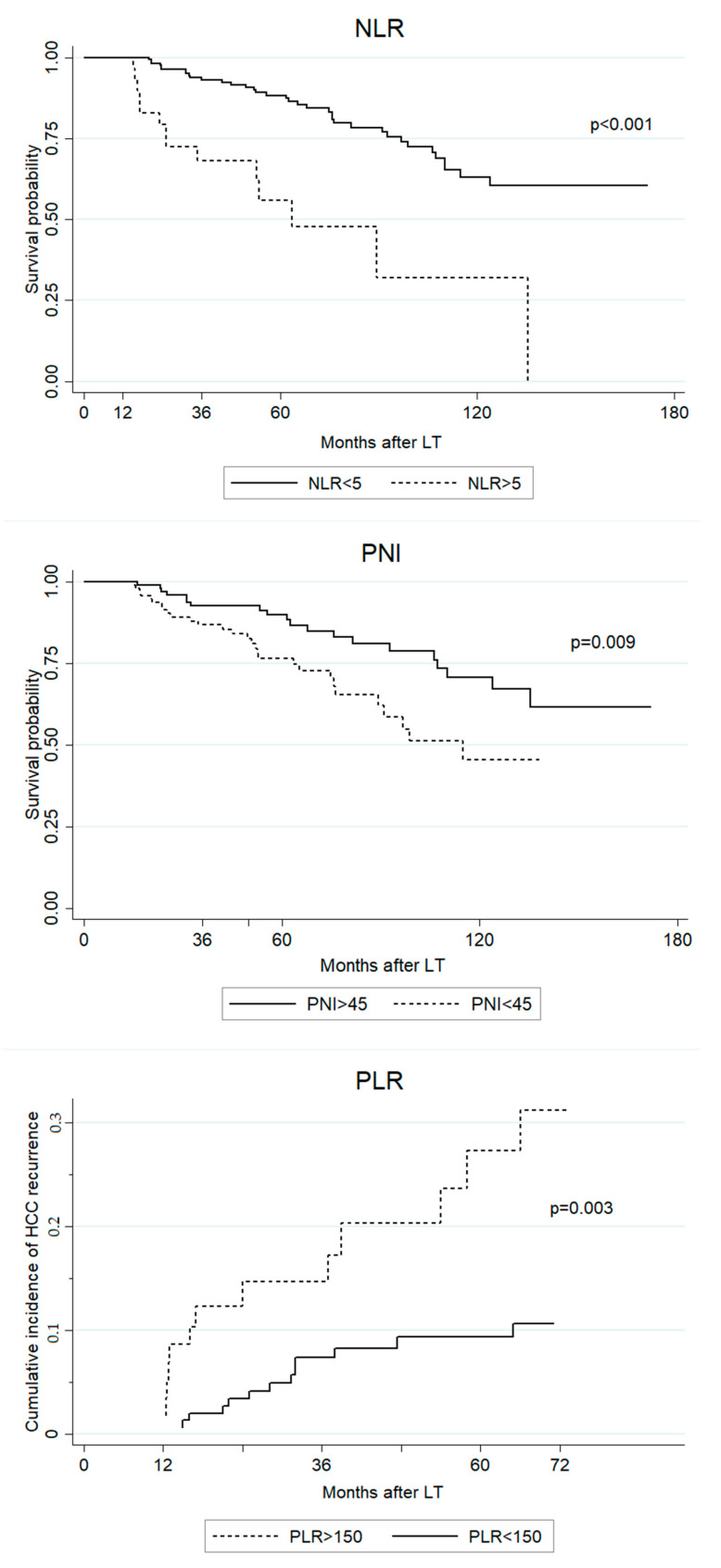
Predictive value of NLR, PNI, and PLR for long-term outcomes, using conventional clinically-oriented cutoffs [2,3,5,6,7,8,11,12].

In univariate analysis, 1-year post-LT PLR, NLR, PNI, and CONUT as well as units of packed blood cells transfused at LT, ml of frozen fresh plasma transfused at LT and HCC recurrence were significantly associated with OS (Table 3). In multivariate analysis, HCC recurrence, 1 year post-LT NLR and 1 year post-LT PNI maintained statistical significance (units of packed blood cells transfused and ml of frozen fresh plasma showed collinearity, therefore only units of packed blood cells transfused was considered in the multivariate model) (Figure 2).

The univariate competing-risk regression analysis showed that 1 year post-LT PLR, NLR and PNI as well as tumor number, tumor maximum diameter, grading, and microvascular invasion were significant risk factors for late tumor recurrence. In multivariate analysis, 1-year post-LT PLR, tumor number, tumor maximum diameter, grading, and microvascular invasion were identified as independent predictors of late HCC recurrence (Table 4) (Figure 2).

### 3.5. Predictive Factors of Pre-LT and 1 Year Post-LT Inflammatory and Nutritional Biomarkers

HCC features (number, max diameter, grading, microvascular invasion) and pre-LT liver disease severity (MELD score, Child–Pugh class) were tested as potential determinants of pre-LT PLR, pre-LT NLR, as well as PLR, NLR, and PNI at 1 year post-LT (Table 5). In the linear regression model, it was found that pre-LT PLR was significantly predicted by tumor grading and tumor number, while pre-LT NLR was predicted by MELD score, Child–Pugh score and tumor grading; both 1-year post-LT PLR and post-LT NLR were significantly predicted by microvascular invasion and pre-LT AFP serum levels. Additionally, 1-year post-LT NLR was also predicted by tumor max diameter. Microvascular invasion and grading were also identified as significant determinants of 1-year post-LT PNI. Pre-LT PNI was not predicted by HCC features but only by pre-LT liver disease severity severity (data not shown).

## 4. Discussion

LT has a radical impact on the immune–inflammatory and metabolic–nutritional status of the recipient. The removal of the chronically inflamed cirrhotic liver and the regaining of normal liver function, with recovery from the complications of liver failure and portal hypertension, contrast with a high surgical invasiveness, potentially severe surgical complications, and immunosuppressant toxicity [13,14]. However, the data assessing the post-transplant outcome in terms of inflammatory state are very limited [1,15], while data on the post-transplant metabolic recovery are extremely heterogeneous, variably reporting a significant increase in the prevalence of metabolic syndrome and an incomplete recovery from pre-LT sarcopenia [16,17,18].

In the present investigation, the early postoperative period was characterized by a significant worsening of the nutritional status of recipients, probably due to the catabolic state induced by surgical stress and inflammatory response, postoperative fasting, and surgical complications. This result was in line with previous studies investigating other malnutrition features, such as sarcopenia [16]. However, in mid/long-term follow-up, CONUT and PNI tended to significantly improve, thus supporting the beneficial effect of LT on nutrition [19]. Moreover, it was shown that nutritional biomarkers did not have any prognostic value as preoperative variables, but actually acquired it as post-transplant ones. It may be speculated that recipients regained a nutritional profile similar to the general population after LT, and those who became malnourished or failed to recover from pre-LT malnutrition despite LT, had an increased risk of early death or tumor recurrence. Of course, obesity, diabetes, and dyslipidemia, rather than undernutrition, currently represent the most frequent and impactful long-term complications after LT, due to the inherent risk of cardiovascular disease and death [18,19]. However the evidence that 1-year post-LT PNI was predicted by HCC features on explanted livers further highlights the pathogenic association between malnutrition, aggressive tumor biology, and risk of tumor recurrence [11,12]. Unfortunately, no other studies in LT recipients are available for comparison with our results. However, the prognostic value of postoperative PNI has been previously explored in an HR setting. In patients with HCC within Milan criteria and hypersplenism, 1-month post-HR PNI was identified as a significant predictive factor for survival and tumor recurrence [20]. Furthermore, a decrease of PNI value at 1 year post-HR, compared to preoperative values, was found as a negative prognostic factor in HBV-positive patients with HCC within Milan criteria [21]. Therefore, in long-term follow up, PNI may be effectively used in clinical practice as a reliable prognostic biomarker. The early identification of those patients who did not benefit from the metabolic curative effect of LT may not only prompt a therapeutic intervention but also warn of an increased risk of HCC recurrence.

On the contrary, the analysis of the postoperative trend of inflammatory biomarkers showed that LT may not sustain a significant improvement of the recipients’ inflammatory status. As a matter of fact, in the present study it was shown that since POM 3, both PLR and NLR leveled off at values that were similar to pre-LT for NLR, and significantly higher for PLR. Parisi et al. [22] measured these inflammatory biomarkers at pre-LT, 1 month and 6 months post-LT in 150 HCC patients within Milan criteria, and found the same postoperative trend with similar median values: PLR, pre-LT 68 (present study 74.1), 6 months post-LT 135 (124.4); NLR, pre-LT 2.2 (2.9), 6 months post-LT 2.5 (2.6). The available data on normal values of PLR and NLR in healthy individuals are quite heterogeneous, probably due to the presence of demographic differences among the study populations. As a matter of fact, race, gender, age, BMI, and smoking have been variably identified as significant determinants of inflammatory biomarkers [23,24]. In a European study [25] on active adults in good health, the mean NLR value was 1.65, while in two Chinese studies [24,26] on healthy adult/old subjects the median NLR value was 1.77 and 1.72, respectively, and the median PLR value was 99 and 108, respectively. In the present study, the median NLR and PLR values at 1 year post-LT were 2.4 and 118.2, respectively, which are indeed higher than the aforementioned reference values. An American study [23] actually reported a mean NLR value of 2.15, but the study population also comprised subjects with comorbidities, such as diabetes, cardiovascular disease, or obesity, which are all associated with chronic pro-inflammatory states [23,27].

In contrast with our results as well as with several previous meta-analyses [1,2,3,4,5,6,7,8], none of the inflammatory biomarkers, either as pre-LT or post-LT variables, were identified as significant predictors of HCC recurrence in the study of Parisi et al. [22]. To be noticed, a competing-risk regression model was not used in such analysis and the potential predictors of OS were not investigated. As already underlined by the Authors, the study population was characterized by relatively favorable HCC features which may have influenced the results. On the contrary, in the present investigation, PLR and NLR not only predicted OS and HCC recurrence as pre-LT variables, but also maintained their prognostic value in the mid/long-term after LT. Similarly, in an HR setting, a significant increase of PLR at 1 month post-HR (compared to preoperative values) was found as an independent risk factor for early death and tumor recurrence in HBV-positive patients with early HCC [28]. Previous studies have already reported a significant correlation between pre-LT inflammatory biomarkers and biological tumor behavior, such as grading and microvascular invasion [1,29]. However our investigation demonstrated that such pathogenic association was maintained even at 1 year post-LT. Such results may enforce the hypothesis that a significant pro-inflammatory state may be actually reactive to a tumor that has already reached the stage of microscopic systemic disease, for whom LT cannot be curative [1,29]. Of course, other determinants of recipients’ post-LT inflammatory status may be implicated, such as graft rejection, immunosuppressant therapies, liver disease recurrence, infections, or metabolic complications [22]. However, it has been extensively demonstrated that chronic inflammation may not only impair an effective immunosurveillance but also act as a primary trigger of tumor progression and metastasis [1,22]. In particular, neutrophil and platelets could promote metastatic dissemination via increased levels of the cytokine vascular endothelial growth factor, angiogenesis-regulating chemokines, and proteases [1,29]. It was interesting to notice that the same independent and specific prognostic value of NLR (OS) and PLR (HCC recurrence) was concordantly identified at 1 year post-LT, in the present study, as well as at pre-LT, in our previous investigation [10]. Such results further enforce the potential role of PLR and NLR in the pre-LT selection of HCC patients.

The precision medicine model, which integrates clinical, molecular, and genetic data to achieve a clinical management that is tailored to the specific characteristics and risks of each patient, is emerging as the most effective health care strategy [30,31]. This is particularly evident in LT candidates with HCC, where the pathogenic interaction among tumor cells, the patient’s immune system and metabolic function, surgical invasiveness, and post-LT immunosuppressant therapies is very tight and has significant implications for long-term outcomes. Biomarkers are the cornerstone of precision medicine, but their identification as well as their real clinical effectiveness depend on the exact understanding of the disease pathogenesis [30,31]. Therefore, further studies will be surely warranted to explore in particular the potential association between inflammatory biomarkers and circulating tumor cells or distant micrometastasis, as well as to identify those post-LT clinical factors which significantly sustain a persistent pro-inflammatory state in LT recipients.

This study presents several limitations: it used a retrospective approach for the data analysis; the immune–inflammatory and metabolic–nutritional status of the patients was investigated only by laboratory biomarkers; the prevalence of metabolic syndrome or pathologic body mass composition features (visceral obesity, sarcopenia), immunosuppressant dosages, post-LT changes of spleen size, and post-LT complications were not specifically investigated.

## 5. Conclusions

LT seems to promote an efficacious recovery and normalization of the nutritional status of recipients, but those who do not benefit from this effect at long-term follow-up are at increased risk of poor survival and HCC recurrence. With this perspective, a routine monitoring of PNI may represent a cost-effective, easy, and prognostically impactful strategy. On the other hand, the effect of LT on the recipient inflammatory status seems limited as in long-term follow-up post-LT PLR and NLR tend to level off at values similar to pre-LT, and maintain the same prognostic value. HCC in LT candidates with high PLR and/or NLR may have a more aggressive biology, requiring these patients to be thoroughly and more carefully assessed in the pre-LT workup. Moreover, NLR and PLR may be also used as reliable prognostic parameters for long-term clinical surveillance.

## Figures and Tables

**Table 1:** Demographic and clinical data on recipients, graft characteristics, and surgical details.

Gender (M:F)	291:33
Age (years)	58 (52–62)
BMI	25.3 (23.2–28.1)
Pre-LT diabetes (%)	54 (16.9%)
HIV positivity (%)	29 (9.0%)
HCV positivity (%)	180 (55.6%)
HBV positivity (%)	70 (21.6%)
Alcohol abuse (%)	96 (29.6%)
MELD score	12 (9–16)
Child–Pugh score (%)	
- A	139 (42.9%)
- B	122 (37.6%)
- C	62 (19.5%)
Pre-LT PLR	74.2 (50.3–108.5)
1 year post-LT PLR	118.2 (79.1–161.5)
Pre-LT NLR	2.9 (1.9–4.8)
1 year post-LT NLR	2.4 (1.7–3.7)
Pre-LT PNI	38.6 (34.5–44.1)
1 year post-LT PNI	45.6 (41.9–49.5)
Pre-LT CONUT	5 (3–7)
1 year post-LT CONUT	2 (1–3)
Pre-LT AFP, (ng/L)	9.8 (4.6–40.2)
Donor age (years)	61.4 (50.1–72.9)
Donor BMI	25.8 (23.7–27.8)
Total ischemia time (min)	470 (401–550)
Packed blood cells transfusion, (UI)	3 (0–7)
Frozen fresh plasma transfusion, (mL)	1000 (0–2000)
Tumor number	2 (1–3)
Tumor max diameter (cm)	2.3 (1.5–3)
Bilobar tumor distribution (%)	82 (25.3%)
Edmonson–Steiner grading (%)	
- Complete necrosis	12 (3.7%)
- G1	54 (16.6%)
- G2	172 (53.1%)
- G3	80 (24.7%)
- G4	6 (1.9%)
Microvascular invasion (%)	50 (15.4%)

AFP: Alpha-Fetoprotein, BMI: body mass index, CONUT: controlling nutritional status, HBV: hepatitis B virus, HCV: hepatitis C virus, HIV: human immunodeficiency virus, HCC: hepatocellular carcinoma, LT: liver transplantation, MELD: Model for end-stage liver disease, NLR: Neutrophil-to-Lymphocyte Ratio, PLR: Platelet-to-Lymphocyte Ratio, PNI: Prognostic Nutritional Index.

**Table 2:** Univariate analysis of prognostic value for mortality and tumor recurrence of inflammatory and nutritional biomarkers, pre- and post-LT, in the whole study population (*n* = 324).

	Mortality			HCC Recurrence		
Factors	HR	95% Conf. Interval	*p*-Val	SHR	95% Conf. Interval	*p*-Val
**PLR**						
pre-LT	1.004	1.002–1.006	**<0.001**	1.005	1.002–1.007	**0.001**
POD 1	1.001	0.999–1.002	0.399	1.001	0.999–1.003	0.196
POD 3	1.001	0.998–1.003	0.354	1.001	0.998–1.004	0.384
POD 5	1.001	0.998–1.004	0.295	0.999	0.996–1.003	0.982
POD 7	1.003	1.001–1.005	**0.012**	0.999	0.996–1.002	0.769
POM 3	1.003	1.001–1.005	**0.003**	1.001	0.998–1.003	0.333
POM 6	1.005	1.004–1.008	**<0.001**	1.003	1.001–1.006	**0.018**
1 year post-LT	1.005	1.003–1.007	**<0.001**	1.003	1.001–1.006	**0.010**
**NLR**						
pre-LT	1.058	1.030–1.087	**<0.001**	1.041	1.003–1.081	**0.036**
POD 1	1.007	0.998–1.016	0.116	1.001	0.988–1.013	0.886
POD 3	1.013	1.001–1.025	**0.032**	0.989	0.964–1.016	0.456
POD 5	1.029	1.013–1.044	**<0.001**	0.977	0.944–1.011	0.187
POD 7	1.054	1.039–1.069	**<0.001**	0.953	0.913–1.001	0.126
POM 3	1.005	1.001–1.011	**0.024**	1.008	1.002–1.016	**0.017**
POM 6	1.210	1.158–1.266	**<0.001**	1.071	1.013–1.133	**0.016**
1 year post-LT	1.058	1.030–1.087	**<0.001**	1.157	1.007–1.331	**0.040**
**PNI**						
pre-LT	0.988	0.962–1.014	0.382	1.018	0.982–1.057	0.315
POD 1	0.952	0.915–1.000	0.145	1.029	0.975–1.086	0.288
POD 3	0.984	0.942–1.027	0.473	0.999	0.943–1.058	0.977
POD 5	0.980	0.941–1.020	0.326	1.006	0.944–1.072	0.840
POD 7	0.956	0.916–0.998	**0.044**	1.009	0.948–1.073	0.776
POM 3	0.943	0.911–0.976	**0.001**	1.005	0.954–1.059	0.834
POM 6	0.911	0.874–0.949	**<0.001**	1.008	0.963–1.054	0.722
1 year post-LT	0.862	0.818–0.908	**<0.001**	0.911	0.844–0.984	**0.019**
CONUT						
pre-LT	0.956	0.876–1.044	0.326	0.936	0.837–1.047	0.251
POD 1	1.071	0.944–1.216	0.282	0.850	0.719–1.005	0.158
POD 3	1.003	0.910–1.106	0.944	0.970	0.850–1.108	0.662
POD 5	1.006	0.902–1.122	0.904	1.001	0.833–1.204	0.987
POD 7	1.158	1.017–1.319	**0.026**	0.959	0.812–1.133	0.628
POM 3	1.211	1.075–1.365	**0.002**	1.002	0.832–1.207	0.980
POM 6	1.335	1.195–1.493	**<0.001**	0.951	0.797–1.135	0.582
1 year post-LT	1.304	1.099–1.546	**0.002**	1.107	0.812–1.508	0.518

CONUT: controlling nutritional status, HR: hazard ratio, LT: liver transplantation, NLR: Neutrophil-to-Lymphocyte Ratio, PLR: Platelet-to-Lymphocyte Ratio, PNI: Prognostic Nutritional Index, POD: postoperative day, POM: postoperative month, SHR: subdistribution hazard ratio, bold numbers mark statistical significance.

**Table 3:** Univariate and multivariate Cox analysis of prognostic factors for OS, in subgroup analysis of patients with overall survival (OS) > 1 year and no early HCC recurrence (*n* = 266).

	Univariate Analysis			Multivariate Analysis		
Factors	HR	95% Conf. Interval	*p*-Val	HR	95% Conf. Interval	*p*-Val
Sex						
- male	1					
- female	0.850	0.340–2.123	0.728			
Age	1.016	0.981–1.052	0.367			
Pre-LT BMI	0.955	0.885–1.031	0.244			
Pre-transplant diabetes	0.870	0.395–1.917	0.730			
HIV positivity	1.115	0.481–2.590	0.798			
HCV positivity	0.892	0.548–1.451	0.646			
HBV positivity	0.870	0.4945–1.533	0.631			
Alcohol abuse	1.523	0.915–2.533	0.105			
MELD score	0.971	0.926–1.018	0.236			
Child–Pugh score	0.839	0.585–1.205	0.343			
Donor age	1.003	0.988–1.018	0.654			
Donor BMI	0.987	0.925–1.054	0.706			
Total ischemia time	1.001	0.999–1.003	0.303			
Packed blood cells transfusion	1.044	1.004–1.085	**0.029**	1.058	1.004–1.117	**0.040**
Frozen fresh plasma transfusion	1.001	1.000–1.002	**0.046**			
Pre-LT AFP	1.000	0.999–1.000	0.277			
HCC recurrence	3.916	2.339–6.556	**<0.001**	5.428	2.859–10.307	**<0.001**
1 year post-LT PLR	1.005	1.002–1.007	**<0.001**			
1 year post-LT NLR	1.332	1.194–1.486	**<0.001**	1.218	1.053–1.408	**0.008**
1 year post-LT PNI	0.863	0.814–0.915	**<0.001**	0.913	0.851–0.978	**0.011**
1 year post-LT CONUT	1.286	1.067–1.550	**0.008**			

AFP: Alpha-Fetoprotein, BMI: body mass index, CONUT: controlling nutritional status, HBV: hepatitis B virus, HCC: hepatocellular carcinoma, HCV: hepatitis C virus, HIV: human immunodeficiency virus, HR: hazard ratio, LT: liver transplantation, MELD: Model for end-stage liver disease, NLR: Neutrophil-to-Lymphocyte Ratio, PLR: Platelet-to-Lymphocyte Ratio, PNI: Prognostic Nutritional Index, bold numbers mark statistical significance.

**Table 4:** Univariate and multivariate analysis of prognostic factors for HCC recurrence, in subgroup analysis of patients with OS > 1 year and no early HCC recurrence (*n* = 266).

	Univariate Analysis			Multivariate Analysis		
Factors	SHR	95% Conf. Interval	*p*-Val	SHR	95% Conf. Interval	*p*-Val
Sex						
- male	1					
- female	2.093	0.844–5.190	0.211			
Age	1.009	0.969–1.059	0.702			
Pre-LT BMI	0.952	0.864–1.051	0.334			
Pre-transplant diabetes	0.336	0.081–1.403	0.135			
HIV positivity	1.042	0.327–3.316	0.944			
HCV positivity	0.565	0.286–1.119	0.202			
HBV positivity	1.917	0.959–3.830	0.165			
Alcohol abuse	1.042	0.502–2.163	0.911			
MELD score	0.939	0.857–1.028	0.177			
Child–Pugh score	0.587	0.346–1.995	0.148			
Donor age	0.994	0.974–1.015	0.622			
Donor BMI	0.931	0.847–1.024	0.143			
Total ischemia time	1.001	0.998–1.003	0.530			
Packed blood cells transfusion	0.994	0.925–1.070	0.891			
Frozen fresh plasma transfusion	0.999	0.999–1.000	0.363			
Pre-LT AFP	1.000	0.999–1.001	0.341			
1 year post-LT PLR	1.004	1.001–1.006	**0.009**	1.005	1.001–1.006	**0.008**
1 year post-LT NLR	1.166	1.009–1.349	**0.038**			
1 year post-LT PNI	0.900	0.830–0.975	**0.011**			
1 year post-LT CONUT	1.165	0.838–1.619	0.364			
Tumor number	1.407	1.203–1.646	**<0.001**	1.504	1.26–1.795	**<0.001**
Tumor max diameter	1.284	1.116–1.478	**0.001**	1.344	1.145–1.578	**<0.001**
Edmonson–Steiner grading	2.250	1.372–3.687	**0.001**	2.030	1.045–3.945	**0.037**
Microvascular invasion	4.859	2.338–10.100	**<0.001**	3.511	1.605–7.681	**0.032**

AFP: Alpha-Fetoprotein, BMI: body mass index, CONUT: controlling nutritional status, HBV: hepatitis B virus, HCC: hepatocellular carcinoma, HCV: hepatitis C virus, HIV: human immunodeficiency virus, LT: liver transplantation, MELD: Model for end-stage liver disease, NLR: Neutrophil-to-Lymphocyte Ratio, PLR: Platelet-to-Lymphocyte Ratio, PNI: Prognostic Nutritional Index, SHR: subdistribution hazard ratio, bold numbers mark statistical significance.

**Table 5:** Linear regression analysis of predictors of PLR, NLR, and PNI.

Factors	Regression Coefficient	95% Conf. Interval	*p*-Val	Regression Coefficient	95% Conf. Interval	*p*-Val	
	**PLR**						
	**pre-LT**			**1 year post-LT**			
MELD score	0.255	−0.959 to 1.471	0.679	0.656	−1.144 to 2.457	0.473	
Child–Pugh score	7.635	−3.347 to 18.619	0.172	−4.916	−20.333 to 10.501	0.530	
Pre-LT AFP	0.001	−0.0136 to 0.016	0.845	0.032	0.013–0.050	**0.001**	
Tumor number	5.408	0.018 to 10.797	**0.049**	0.256	−7.296 to 7.810	0.947	
Tumor max diameter	0.645	−5.470 to 6.760	0.836	1.799	−5.061 to 8.660	0.606	
Edmonson–Steiner grading	10.108	1.162 to 21.378	**0.050**	9.166	−6.697 to 25.031	0.256	
Microvascular invasion	13.810	−9.167 to 36.789	0.238	36.989	3.763 to 70.215	**0.029**	
	**NLR**						
	**pre-LT**			**1 year post-LT**			
MELD score	0.196	0.115 to 0.277	**<0.001**	−0.005	−0.053 to 0.041	0.816	
Child–Pugh score	2.095	1.368 to 2.822	**<0.001**	0.071	−0.344 to 0.486	0.736	
Pre-LT AFP	−0.000	−0.000 to.008	0.928	0.081	0.035 to 0.132	**0.002**	
Tumor number	0.172	−0.205 to 0.549	0.370	0.054	−0.146 to 0.255	0.595	
Tumor max diameter	−0.367	−0.790 to 0.055	0.089	0.185	0.007 to 0.364	**0.042**	
Edmonson–Steiner grading	0.836	0.051 to 1.621	**0.037**	0.286	−0.128 to 0.701	0.175	
Microvascular invasion	0.887	−0.728 to 2.503	0.281	1.125	0.252 to 1.999	0.012	
	**PNI**						
				**1 year post-LT**			
MELD score				−0.022	−0.153 to 0.107	0.729	
Child–Pugh score				−0.405	−1.539 to 0.729	0.482	
Pre-LT AFP				−0.000	−0.002 to 0.000	0.226	
Tumor number				−0.218	−0.763 to 0.326	0.431	
Tumor max diameter				−0.022	−0.519 to 0.473	0.927	
Edmonson–Steiner grading				−1.345	−2.463 to −0.228	**0.019**	
Microvascular invasion				−3.785	−6.142 to −1.427	**0.002**	

AFP: Alpha-Fetoprotein, CONUT: controlling nutritional status, LT: liver transplantation, MELD: Model for end-stage liver disease, NLR: Neutrophil-to-Lymphocyte Ratio, PLR: Platelet-to-Lymphocyte Ratio, PNI: Prognostic Nutritional Index, bold numbers mark statistical significance.

## Data Availability

The data presented in this study are available on specific and motivated request to the corresponding author. The data are not publicly available due to privacy restrictions.
